# Supplementary material for: Frontal and Cerebellar Atrophy Supports FTSD-ALS Clinical Continuum
Source: Front Aging Neurosci. 2020 Nov 26;12:593526. doi: 10.3389/fnagi.2020.593526 (PMC7726473; doi:10.3389/fnagi.2020.593526)
Supplement: Supplementary file 1 [file Data_Sheet_1.pdf]

Supplementary material: VBM and $\overline{CT}$.

Methods

Overlap between atrophy and resting state networks (RSN)

In order to assess the potential functional implications of the atrophic areas, we classified all altered voxels based on a >10% spatial overlap between clusters of volume reductions, in patients (FTSD, FTSD-ALS, ALS) with respect to CTR, and standard resting state networks (RSNs) (Castellazzi *et al.*, 2018) .

Correlation between volume and $\overline{CT}$

SPM12 was used to perform multiple regression analyses on all subjects to correlate GM and WM volume with $\overline{CT}$ values. For each neuropsychological score, a multiple regression analysis was performed on all patients considered together to determine possible areas responsible for the distribution of results.

Results

Overlap between atrophy and resting state networks

Our approach allowed us to locate atrophic structures with respect to standard RSNs. Atrophic areas in the ALS group were predominantly located in standard areas of the sensory motor network (SMN); atrophy of FTSD-ALS overlapped areas belonging not only to motor (e.g. frontal cortex (FCN)) but also to other sensory networks (e.g. the occipital visual network (OVN)), several higher-functions, as well as hippocampal areas belonging to the default mode network (DMN); atrophy in FTSD patients involved standard areas of almost all functional systems, with a further extensive involvement of the DMN and the cerebellar network. The location of all abnormal voxels with reference to RSN involvement is shown in Table S1.

Correlation between volume and $\overline{CT}$

Correlations between volume and whole brain $\overline{CT}$ in CTR, all patients and different patient groups are reported in the supplementary material Table S2.

In CTR, positive correlation was found between $\overline{CT}$ and volume of several GM regions of the frontal, parietal and temporal lobes.

Considering all patients, lower $\overline{CT}$ correlated with lower GM volume of areas in the frontal, parietal and temporal lobes, and with WM volume of regions connecting frontal and temporal/occipital lobes.

In the FTSD group, lower $\overline{CT}$ correlated with lower GM volume of areas in the parietal lobe, whereas correlation with volume of WM regions connecting frontal and temporal/occipital lobes emerged lowering the statistical threshold.

In the ALS group, lower $\overline{CT}$ correlated with lower GM volume in the cerebellum, while lowering the statistical threshold also WM regions connecting frontal and temporal lobes are involved.

Table S1: Overlap between regions of volume reduction and resting state networks

GROUP	MVN	CBL	OVN	SMN	DMN	AN	SN	LVN	LVAN	ECN	WMN	FCN	RVAN
**ALS**				×		×							
**FTSD-ALS**			×		×	×			×	×	×	×	
**FTSD**	×	×	×	×	×	×	×		×	×	×	×	×

Resting state networks (RSNs) involved by atrophy for each group of patients (rows) compared to controls. "×" indicates areas with reduced volume overlapping with at least 10% of voxels belonging to specific RSNs. MVN = Medial Visual Network; CBLN = Cerebellar Network; OVN = Occipital Visual Network; SMN = Sensory Motor Network; DMN = Default Mode Network; AN = Auditory Network; SN = Salience Network; LVN = Lateral Visual Network; LVAN = Left Ventral Attention Network; ECN = Executive Control Network; WMN = Working memory Network; FCN = Frontal Cortex Network; RVAN = Right Ventral Network.

Table S2: Correlation of local voxel brain morphometry (VBM) properties with whole brain cortical thickness ($\overline{CT}$)

Brain region		CTR	PT	FTSD	ALS
	Superior frontal gyrus	L	L	R *	
	Medial frontal gyrus	L	BIL	R *	
	Inferior frontal gyrus		BIL		
	Rectum gyrus		L		
	Paracentral lobule	R	L		
	Insula		L		
**Gray matter**	Fusiform gyrus	BIL	L		
	Parahippocampal gyrus	R	L		
	Superior temporal gyrus	R	BIL		
	Medial temporal gyrus	BIL	BIL		
	Inferior temporal gyrus	L	BIL		
	Hippocampus		L		
	Cingulate cortex	R	L	L *	

	Postcentral gyrus		L		
	Superior lobule	R			
	Inferior lobule	R	R	R	
	Precuneus	R	L		
	Supramarginal gyrus	R	R	R	
	Lingula	R	R		
	Middle occipital gyrus	L	L		
	Superior orbital frontal gyrus			R *	
**White matter**	Inferior fronto-occipital fascic		L	L *	L *
	Forceps minor/Major			(Maj) L*	(min) R*
	Cingulum		L		
	Superior longitudinal fascic		L	L *	
	Inferior longitudinal fascic		L		R *
	Uncinate fasciculus				L *
**Cerebellum**	Lobule VII				R
	Lobule IX				BIL

Regions of correlation among CT score and cerebral volume in controls (CTR), all patients (PT), and each group of patients (FTSD, FTSD-ALS, ALS). Significance was set at p=0.05 FWE corrected at cluster-level. (*) indicates exploratory results at p=0.001 with a minimum cluster extent of k=160 voxels. The lateralization is identified with: L = left; R = right; BIL = bilateral.

Discussion

The overlap of atrophy results and resting state networks was performed with RSN extracted from data acquired on a different cohort of healthy subjects, which were then used as templates. Future work could consider RSN changes in concomitance of atrophy changes in the same population.

These are exploratory results (p<0.001, uncorrected) except for the results in the ALS group, it is intriguing to consider $\overline{CT}$ correlations with GM and WM volume in each group. Indeed, the different etiology of these patients brings out some differences in morphological changes that could subtend $\overline{CT}$ and VBM volumes correlations. In particular, in ALS, $\overline{CT}$ correlates with volume in the posterior cerebellum, and in particular area VIII and IX, known to be key to motor control, as well as motor learning and sensory integration. This is different from previous studies that showed $\overline{CT}$ correlations with the precentral cortex, cingulum and insula.[31,32] In our cohort, though, $\overline{CT}$ correlated with extensive WM areas affecting long tracts connecting main cortical lobes, including the forceps minor connecting interhemispheric frontal cortices, the inferior longitudinal fasciculus connecting temporal and occipital lobes, the UF connecting the

limbic system to the temporal and frontal lobes and the inferior fronto-occipital fasciculus connecting occipital and frontal cortices. These correlations that emerge only in ALS patients, suggest that white matter integrity has a key role in preserving cortical cell density, as measured through $\overline{CT}$. Indeed, no correlations were found in FTSD-ALS, while CT and volume correlated in a number of frontal lobe GM regions and in only temporal WM in FTSD, supporting the different clinical presentation of these patients' groups.
